# Leaf wrinkling in *Brassica campestris*: involvement of brassinosteroid signaling

**DOI:** 10.1093/plphys/kiaf446

**Published:** 2025-09-27

**Authors:** Kumari Billakurthi

**Affiliations:** Assitant Features Editor, Plant Physiology, American Society of Plant Biologists; Department of Plant Sciences, University of Cambridge, Cambridge CB2 3EA, UK

Leaves of land plants play a critical role in sustaining life on Earth by contributing to food production and replenishing oxygen in our atmosphere. Through photosynthesis, leaves fix atmospheric carbon dioxide into organic compounds, including sugars, and release oxygen. Leaves also release water vapor into the atmosphere through transpiration. Leaf morphology varies greatly across plant species (Runions et al. 2017), influencing total leaf area, photosynthetic efficiency, and transpiration rates (Vuolo et al. 2016). Moreover, in leafy vegetables, leaf shape significantly affects texture, taste, and attractiveness, thereby influencing consumer preferences and market value (Souza et al. 2021). *Brassica campestris*, commonly known as turnip, is a widely cultivated leafy vegetable that exhibits considerable variation in leaf morphology, including flat and wrinkled leaf types. Turnip thus is a good model species for investigating the molecular mechanisms underlying the leaf shape diversity. Importantly, the degree of leaf wrinkling is a key trait used to classify *B. campestris* varieties. However, the molecular basis of this important trait has remained largely unexplored.

In this issue of *Plant Physiology*, Chen and coauthors (Chen J et al., 2025) investigated the molecular mechanisms behind leaf wrinkling in *B. campestris*. They studied 2 cultivars, “Suzhouqing (“SZQ”) and “Huangya14 (“HY14”), which have flat leaves and wrinkled leaves, respectively. Leaf morphology was assessed at 7, 14, 21, and 120 days after sowing (DAS). While both cultivars initially displayed flat leaves, by 14 DAS, “HY14” began to show wrinkling at the leaf tips. Anatomical analysis revealed that “HY14” exhibited indistinct mesophyll cell layers with large intercellular space at all examined stages (7, 14, and 21 DAS). Notably, “HY14” had more epidermal cells than “SZQ” at 7 DAS but not at later stages. These findings suggest that the developmental program leading to the wrinkled leaf phenotype is established during early leaf development, likely before 7 DAS.

Transcriptome profiling of the two cultivars at 7, 14, and 21 DAS uncovered many differentially expressed genes between them. Notably, genes involved in leaf polarity, epidermal development, and brassinosteroid (BR) signaling pathways were differentially expressed. BZR1, a key transcription factor in the BR signaling pathway, was significantly upregulated in “HY14.” “HY14” also had elevated levels of endogenous brassinolide, which is the active form of BR and is known to promote cell division (Grove et al. 1979).

To further elucidate the role of BR signaling in leaf wrinkling, the authors treated “HY14” with brassinazole, a BR biosynthesis inhibitor. The treatment led to reduced leaf wrinkling and anatomical features that were similar to “SZQ.” The authors verified the transcript levels of *BZR1*, and leaf polarity, cell cycle genes such as *PHABULOSA* (*PHB*), CycD3;1 and CycA2;3 by reverse transcription quantitative PCR, in response to the brassinazole treatments. Mainly, the expression of *BZR1*, *PHB*, and *CycD3;1* were downregulated by the treatments. In contrast, exogenous application of brassinolide to “SZQ” plants induced leaf wrinkling, suggesting that BR signaling contributes to the development of wrinkled leaf phenotype (Chen J et al., 2025). *PHB* is known to control adaxial-abaxial polarity in Arabidopsis (McConnell et al. 2001). Abaxial leaf fates were transformed into adaxial leaf fates in the dominant *phb* mutant line (McConnell et al. 2001). *CycDs* positively regulate the cell divisions (Kono et al. 2007). In wrinkled leaves of “HY14” palisade and sponge mesophyll tissues were indistinguishable and had more epidermal cells. Therefore, the authors hypothesized that *BcBZR1* mediates leaf wrinkling by regulating the expression of *PHB* and *CycD* (Chen J et al., 2025).

To further functionally test this model, the authors overexpressed *BcBZR1* in the flat-leafed cultivar “YQ49.” BcBZR1 overexpression induced leaf wrinkling and the associated anatomical changes. Moreover, transcript levels of *PHB* and *CycD* were upregulated. Conversely, knockdown of *BcBZR1* in “HY14” resulted in flatter leaves, which had more organized mesophyll structure, fewer epidermal cells, and downregulation of *PHB* and *CycD*. These results further strengthened the hypothesis that BcBZR1 might activate the expression of *PHB* and *CycD* and thus induce changes in leaf morphology and anatomy. The authors showed directing binding of BcBZR1 to the promoter regions of *PHB* and *Cyc-D* by chromatin immuno-precipitation quantitative PCR assay and activation of those genes by a dual-luciferase reporter assay, confirming that BcBZR1 mediates leaf wrinkling by directly activating the expression of *PHB* and CycD.

**Figure 1.**
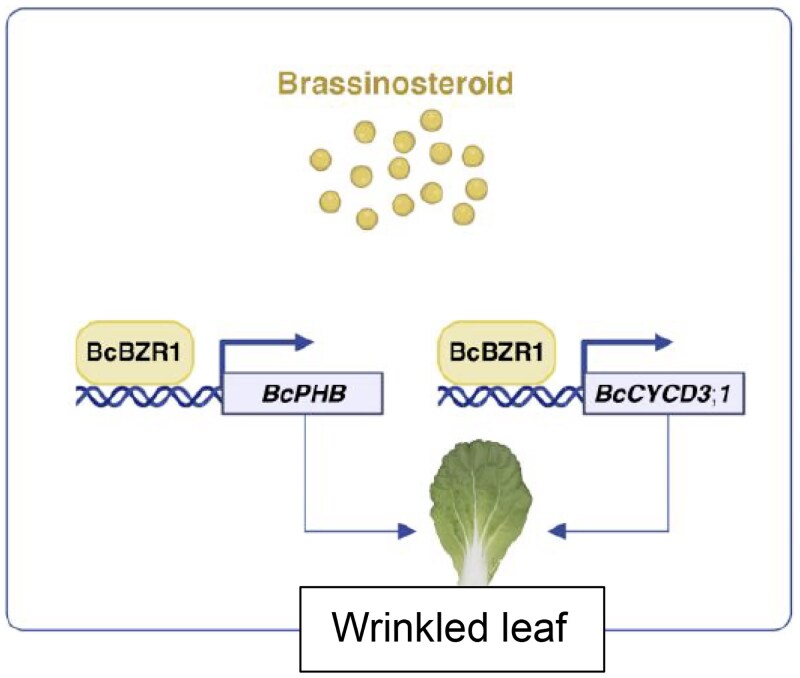
In response to high levels of BR, BRZ1 transcription factor directly activates the expression of *PHB* and *CycD* genes and thus promotes the leaf wrinkling phenotype in *B. campestris* (adapted from Chen et al. 2025).

Collectively, Chen et al. 2025 demonstrate that BR signaling, mediated through BcBZR1, plays a central role in regulating leaf wrinkling in *B. campestris* by modulating the expression of genes involved in cell division and leaf polarity (Fig. 1). Future research could explore how environmental factors modulate this developmental program and how altered leaf morphology impacts photosynthetic performance.
